# Molecular and pharmacodynamic interactions between caffeine and dopaminergic system

**Published:** 2014

**Authors:** M Voiculescu, I Ghiță, A Segărceanu, I Fulga, O Coman

**Affiliations:** * Department of Pharmacology and Pharmacotherapy, Faculty of Medicine, “Carol Davila” University of Medicine and Pharmacy, Bucharest

**Keywords:** caffeine, dopamine, adenosine, Parkinson, schizophrenia

## Abstract

Many drugs targeting dopaminergic system were developed for treating schizophrenia (antagonists of D2 dopaminergic receptors, e.g. antipsychotics) or Parkinson’ disease (agonists of dopaminergic receptors, e.g. L-DOPA). Because many of the patients treated with these drugs consume caffeine based beverages, pharmacodynamics and pharmacokinetics interactions between caffeine and dopaminergic system or drugs influencing this system are possible. The present review is assessing the current available scientific data on pharmacodynamics interactions between the dopaminergic and adenosinergic system but also on caffeine and dopaminergic system interactions. Caffeine can significantly improve Parkinson’s disease symptoms but also the extrapyramidal syndrome induced by antipsychotics via dopaminergic pathways. No study so far has directly evaluated the influence of caffeine in schizophrenia, but there is growing evidence that adenosine dysfunction may contribute to the neurobiological and clinical features of schizophrenia. Caffeine has also effects on the reward system but it seems that this effect does not involve dopaminergic system. Caffeine has some endocrine effects via dopaminergic system such as decreasing the milk production in lactating women or other potential reproductive and nutritional consequences.

Many drugs targeting dopaminergic system were developed for treating schizophrenia (antagonists of D2 dopaminergic receptors, e.g. antipsychotics) or Parkinson’ disease (agonists of dopaminergic receptors, e.g. L-DOPA). Because many of the patients treated with these drugs consume caffeine based beverages, pharmacodynamics and pharmacokinetics interactions between caffeine and dopaminergic system or drugs influencing this system are possible.

**1. Dopamine system in the human brain**

The human brain contains a number of about 300,000 to 400 000 dopaminergic neurons. These neurons in the vast majority are found in three anatomical structures: substantia nigra - pars compacta which is found in the midbrain, the ventral tegmental area and arcuate nucleus. These three structures present afferent and efferent neurons from and to neostriatum, cerebral cortex, hypothalamus and limbic systems. All these neural pathways were first viewed through a fluorescence staining method by Dahls Trom and Fuxe in 1964 [**1**].

Dopamine has many physiological roles as involvement in neuronal pathway of reward, in voluntary movement and secretion of hormones. In terms of pathology, dopamine is incriminated in the appearance of two major neuropsychiatric disorders such as schizophrenia entities in which there is an overactive dopaminergic system and Parkinson's disease there is a reduction of dopamine due to a depletion of dopaminergic neuronal transmission. Dopaminergic transmission pathways are involved both in disease processes but also in the extrapyramidal effects of neuroleptic medication.

**1.1. Structure and synthesis of dopamine**

Dopamine (3,4 - dihydroxyphenylalanine) is an endogenous molecule that is part of the catecholamines system together with epinephrine and norepinephrine. It consists of two benzene rings with two hydroxyl groups thus forming the nucleus of catechol and an amino-ethyl radical attached to the catechol nucleus. 

Tyrosine hydroxylase has the most important role in dopamine synthesis and also in limiting the synthesis and maintaining an adequate dopamine concentration in the brain. The enzymatic activity of this enzyme is done both on short and long term: first by negative feed-back controlled by intracellular dopamine concentration and second by genetic regulation of transcription. 

The second enzyme, DOPA-decarboxylase has a high activity by transforming very fast DOPA in dopamine. There are already available drugs that can influence the activity of this enzyme like carbidopa and benserazide who inhibit DOPA-decarboxylase and are successfully used in treating Parkinson’disease [**2**].

**Fig. 1:**
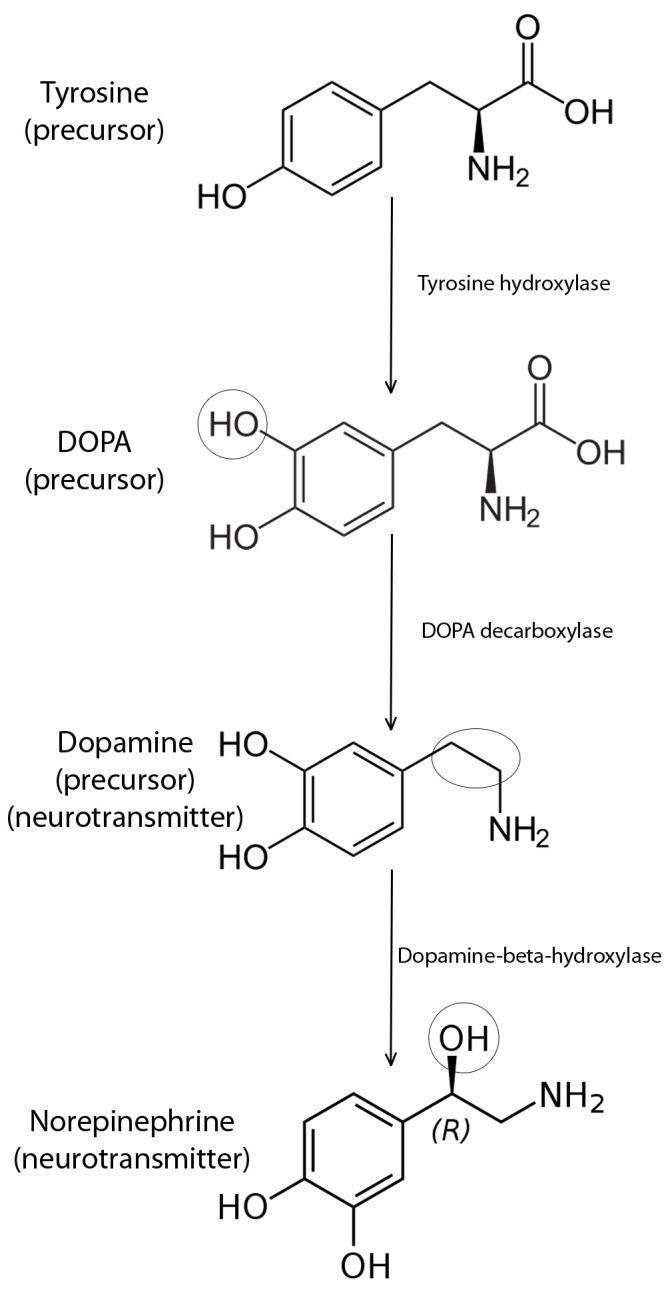
Dopamine synthesis

**1.2. Dopamine catabolism**

Dopamine is metabolized by 2 enzymes: MAO (mono-amine oxydase) with 2 izoenzymes (MAO-A and MAO-B) and COMT (cathecol-ortho-methyl-transferase) with the following metabolites being synthesized: DOPAC (3,4-Dihydroxyphenylacetic acid) and HVA (Homovanillic acid) by MAO and 

3-Methoxytyramine by COMT.

**1.3. Dopaminergic receptors**

These receptors are found in the central nervous system (CNS) at both presynaptic and postsynaptic level. Their first classification was made after a series of biochemical and pharmacological criteria by Kebabian and Calne in 1979 and they were divided into two broad categories: D1-like receptors (D1 and D5) and D2-like receptors (D2, D3 and D4). Whatever the category, these receptors exert their action through G proteins. These receptors contain a transmembrane domain, a residue of aspartic acid and 2 serine residues which represent the binding sites of amino and hydroxyl groups of dopamine [**3**].

D1-like receptors are associated with Gs proteins which stimulate adenylate cyclase activity, in contrast with D2-like receptors which are coupled with Gi proteins that inhibit the activity of the same enzyme. Between the two types of receptors there are differences in terms of drug pharmacodynamics: D1-like receptors have low affinity for some antipsychotics such as sulpiride and affinity for benzodiazepines such as SCH-23390, whereas the D2-like receptors show a high affinity for antipsychotic agents and are also correlated with inhibition of prolactin release from the anterior pituitary gland.

Molecular cloning of these receptors has revealed the existence of two isoforms for both D2 and D3 receptors. In the D2-like class there are differences in the affinity for different drugs. D3 receptors show high affinity for atypical neuroleptics and for dopamine auto-receptors inhibitors [(+) UH232 (+) AJ76] and D4 receptors show high affinity for clozapine [**4**].

**1.4. Dopamine physiological functions**

The role of dopamine in both normal behavior and in pathology was an object of study since the moment there has been observed a direct link between this neurotransmitter and Parkinson's disease, schizophrenia, motivation and reward mechanisms.

The increase of dopaminergic transmission at least up to a certain rate will cause an activation of motor behavior (locomotion) and on the other hand a decrease of dopamine will lead to akinetic syndromes including "extreme muteness " [**5**, **6**].

Over a certain dopaminergic transmission rate of growth, will produce a motor behavior in laboratory animals known as "stereotypes", which are repeated constant movements and whose human equivalent is the compulsive behavior [**7**]. A practical example of the effect of dopamine on stimulation of motor activity is represented by quinpirole, a dopamine agonist selective D2 and D3 receptors which produces an increase of up to six times the distance traveled in the open field test [**8**]. At a normal alertness, dopamine will stimulate: exploration behavior more than social behavior, anticipatory behavior more than consumption, sexual activity more than hunger, staccato movements of the eyeballs more than simple motion tracking, up movements of the eyeballs more than down movements.

Dopamine deficiency in experimental animals showed that they lose the ability for spatial orientation and of analogies in outer space [**8**,**9**]. This contrasts the effects of dopamine agonists which stimulate analytical behavior for details of outer space. Only the laboratory animals with a normal level of dopamine will retain the spatial orientation based on new details, though engaged in certain activities such as feeding behaviour [**10**].

The role of dopamine in our projection in time and especially in the future is revealed by the implication of this neurotransmitter in predicted reward. Schultz et al in 1997 determined that during learning by reward, ventral tegmental neurons and substantia nigra neurons show an increased activity. These dopaminergic neurons are stimulated in the learning process only if it is associated with a reward, lack of reward causing inhibition of activity in the two structures [**11**]. Another example of the involvement of dopamine in reward projected in the future is that in both rats and mice, antipsychotics demonstrated a desire to get a quick reward and not a delayed one. Damage to the prefrontal dopaminergic ventral-medial regions and temporal regions has led to what is called "future myopia" [**12**, **13**, **14**, **15**].

A number of researchers have interpreted the involvement of dopamine in male sexual behavior and in other pleasant activities as a stimulation of hedonistic behavior in general. Berridge and Robinson in 1998 underlined that the difference between really getting the pleasure from a reward and the desire to obtain the reward are different. Pleasure in itself implies a positive emotional activity, while the desire to obtain a reward involves a motivational drive that leads to it, so it just has the potential to cause a positive emotional activity or not. This motivational drive is mediated by dopaminergic system and an example of this is being dependent on gambling or drugs for which the payoff can be insignificant, but the process of obtaining it can be very important. This involvement of dopamine in motivational drive may explain why in the pathology of obsessive-compulsive disorders we are dealing with an excessive motivational drive which ultimately leads to achieving goals that do not have any emotional benefit [**16**].

Dopamine appears to be involved in feeling of emotional detachment that was associated with increased levels of this mediator and with certain genes which code D2 dopamine receptors. This feature of emotional detachment is specific to individuals who have a highly developed desire to achieve their goals.

The role of dopamine in intelligence extends beyond involvement in motivational drive, dopamine being involved in at least six areas associated with cognitive intelligence and these cognitive domains are related to outer extra-personal space. Thus, first, it should be noted that reducing the amount of dopamine in the prefrontal cortex is thought to be the cause of memory disruption and of dis-executive behavior of phenylketonuria [**17**]. Dopamine seems to be related to the analytical and executive intelligence in humans. Dopamine system is correlated with dementia in elderly, the low number of dopamine receptors appears to be responsible for the lack of mental flexibility and low scores on cognitive tests.

Dopamine plays a central role in various diseases such as schizophrenia (where dopamine is raised in ventromedial mezo-limbic), Parkinson's disease (where dopamine in the striatum is low), bipolar disorder (dopamine is increased in the manic phase), ADHD (dopamine is reduced in the lateral prefrontal cortex as compared to ventromedial system). The involvement of dopamine is varying in pathogenic mechanisms of these diseases which present a range of different signs and symptoms determined by dopamine and other neurotransmitters. All these conditions have, however, a common deficiency: reduction of executive intelligence (an impairment of planning, working memory, changing strategies) [**18**].

**2. Interactions between dopamine and adenosinergic system**

**2.1. A1A receptors and dopamine D1 receptors interactions**

In 2011 a review done by Ciruela F et al. presented the modulatory effects of adenosine on dopamine systems in view of their relevance to human pathology such as schizophrenia and Parkinson's disease. Adenosine can inhibit several effects of dopamine in the cerebral cortex and basal ganglia. In rat models of Parkinson's disease, for instance, it was shown that nonselective adenosine receptor antagonists (i.e. either caffeine or theophyllamine) enhanced the effect of L-DOPA and other dopamine receptor agonists increasing motor activity. Similarly, it was also described in unilaterally 6-OH-dopamine (which specifically destroys the nigrostriatal dopamine pathway) lesioned rats that the A1 receptors selective antagonist CPT enhanced the motor activating effects of the D1 receptor selective agonist SKF 38393, while the A1 receptor selective agonist CPA counteracted SKF 38393-induced grooming behavior. Hence, in order to explain the results obtained in a large number of behavioral studies, it was suggested that antagonistic adenosine/dopamine interactions were, at least in part, caused by an intramembrane interaction between specific subtypes of dopamine and adenosine receptors, in this case A1 receptors and D1 receptors [**19**].

The above-commented results may then explain the well-documented antagonistic A1 receptor/ D1 receptor interactions found in the neuronal networks of the brain, since they indicate that A1 receptor/D1 receptor heteromerization could determine changes in the pharmacological profile of the receptors. For instance, activation of the A1 receptor could modulate D1 receptor signaling. One of the mechanisms that may explain this action is based on the ability of A1 receptor activation within the heteromer to change the binding characteristics of dopamine to the D1 receptor [**19**].

Activation of A1 receptors reduced the proportion of D1 receptors in the high-affinity state without changing the dissociation constants of the high- and the low affinity binding sites. It was reported that the adenosine A1 receptor selective antagonist DPCPX had an effect on the Ki value of the D1 receptor selective agonist SKF 38393 in A1R/D1R transfected cells, thus adenosine would exert a tonic inhibition of dopamine in the D1 receptor mediated function through the A1R/D1R complex [**19**].

Pretreatment with an A1 receptor agonist caused co-clustering (co-aggregation) of A1 receptors and D1, which was blocked by combined pretreatment with A1 and D1 receptor agonists. These results indicated that the movement of the heteromer and/or clusters of heteromers in the plasma membrane could be agonist-dependent, and that receptor trafficking may depend on the activity of the two receptors of the heteromer [**19**].

The A1 receptor / D1 receptor interaction in this heteromer is relevant for acute A1 receptor antagonism of D1 receptor signaling and for long-term antagonism of A1 receptor to D1 receptor signaling to the Gs protein. 

**2.2. A2A receptors and dopamine D2 receptors interactions**

It is well accepted that the A2A receptor plays a modulatory role in the CNS in general and in the striatum in particular, and that this A2A receptor-mediated neuromodulatory role is somehow related to the ability of this receptor to heteromerize with other GPCRs (G-protein coupled receptors). Indeed, in the last decade the existence of a direct receptor – receptor interaction between A2A receptors and D2 receptors has been demonstrated. Thus, it has been postulated that while the previously described A1 receptor/D1 receptor interaction would modulate the function of the GABAergic dynorphynergic neurons, the A2A receptor/D2 receptor interaction would modulate the function of the GABAergic enkephalinergic neurons [**19**].

The A2A / D2 receptor interaction takes place in both the somatodendritic area and in the nerve terminals of the GABA-ergic enkephalinergic neurons [**20**, **21**, **22**, **23**].

It has been shown that there exists a strong tonic activation of D2R blocking the ability of the A2A receptor to signal through the cAMP-PKA pathway. In this way, in vivo administration of a D2 receptor selective antagonist in the rodent striatum produces a significant increase in the expression of c-fos and preproenkephalin genes, which depends on the ability of the D2 receptor to tonically block A2A receptors signaling activated by endogenous adenosine [**24**]. Overall, two reciprocal antagonistic A2A receptor/D2 receptor interactions have been described, namely an inter-membrane interaction in which the A2A receptor mediates the inhibition of the D2 receptor, thus modulating neuronal excitability and neurotransmitter release, and an interaction at the level of adenylyl-cyclase in which the D2 receptor inhibits A2A receptor-mediated protein phosphorylation and gene expression [**19**]. Novel therapeutic approaches for Parkinson's disease (Istradefylline, Preladenant, Vipadenant) are being developed taking into account not only the particular characteristics of the receptors within the heteromer, but also the fact that the pathology may induce changes in such receptor–receptor interactions involved in the function of neuronal networks of the brain, such as in the basal ganglia and prefrontal cortex.

**3. Caffeine and dopaminergic system**

**3.1. Caffeine’s mechanisms of action**

The adenosine A1 and A2A receptors have high affinity for adenosine and are those responsible for tonic actions of endogenous adenosine [**25**].Caffeine causes most of its biological effects via antagonizing all types of adenosine receptors (ARs). When acting as an AR antagonist, caffeine, used acutely, is doing the opposite of activation of adenosine receptors, due to removal of the adenosinergic tonus. 

**Fig. 2:**
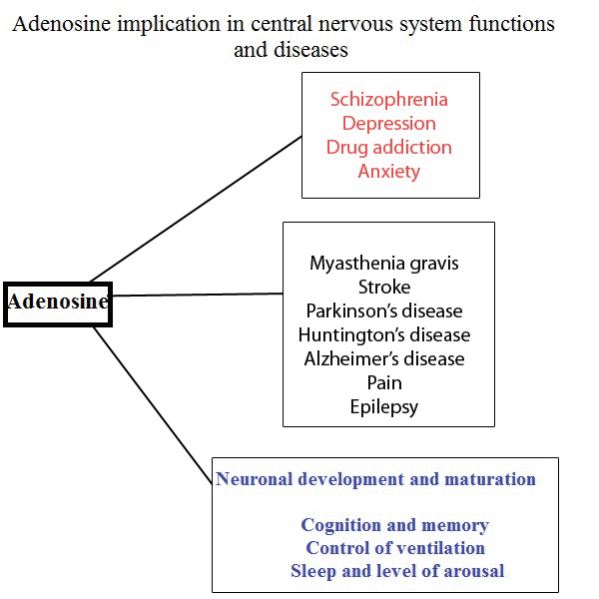
Actions proposed for adenosine on the central nervous systems.

As a pharmacological tool, caffeine is not very useful since its affinity for ARs is low and its selectivity towards the different ARs is also very poor. Caffeine is an antagonist of all subtypes of ARs, and chronic or acute intake of caffeine may affect ARs in different and even opposite ways. Having similar affinity for A1 and A2A Rs [**1**], acute caffeine actions at a given brain area will reflect the preponderant AR activation in that area, since most of the adenosinergic tonus is exerted through that receptor.

The mechanisms mediating caffeine central effects are implicated in the conditioned drug effects induced by dopaminergic drugs although, paradoxically, they appear to be disassociated from dopaminergic mechanisms. While substantial evidence suggests that caffeine stimulant effects are mediated by adenosine antagonism, the central effects of caffeine are complex and diverse [**25**]:

1) inhibition of phosphodiesterases (PDEs) (e.g., PDE1, PDE4, PDE5). These effects (up to 40 % inhibition of phosphodiesterases), according to Daly (2007) [**26**], are observed in concentrations well below those that cause toxic effects. In relation to PDE inhibition, it is interesting to note that caffeine, being a PDE5 inhibitor, operates through a mechanism also used by sildenafil, which is a vasodilator, via selective PDE5 inhibition. So, the potential effects related to these actions need to be investigated to see whether consequent vasodilation might contribute to net caffeine effects. 

2) Promotion of calcium release from intracellular stores. Application of caffeine-halothane contracture test in the diagnosis of malignant hyperthermia is an example of application of this effect.

3) Interfering with GABA-A receptors [**26**].

**Fig. 3:**
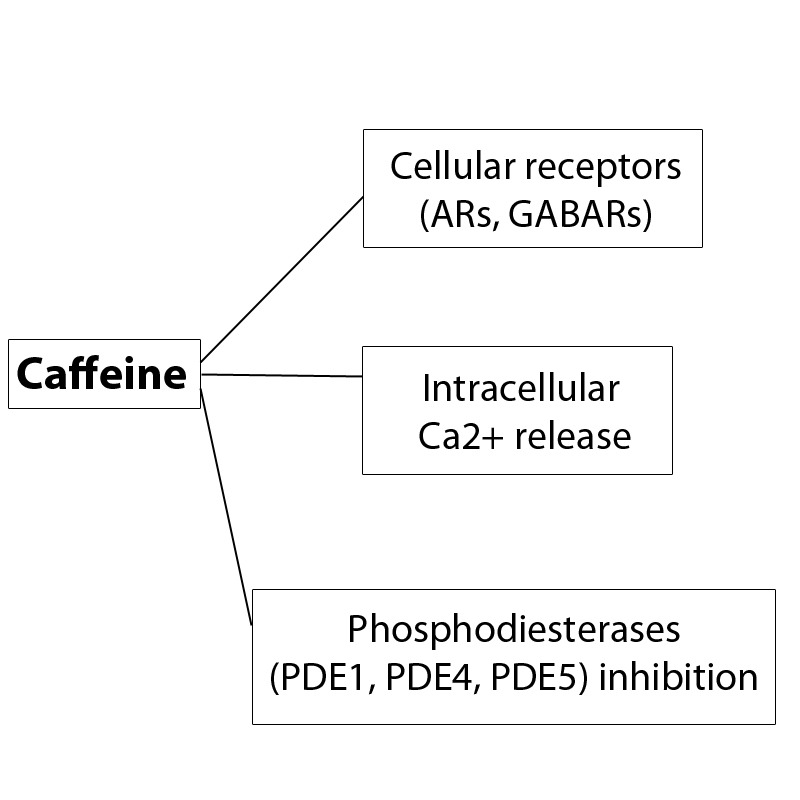
Mechanisms of action of caffeine.

**3.2. Caffeine and Parkinson’s disease**

As shown by numerous studies, caffeine can determine an improvement of Parkinson’s disease symptoms such as catalepsy and muscular rigidity. It has been proposed that while both A1 and A2A antagonism contribute to caffeine's motor stimulatory effects, A1 antagonism may play a greater role when administered acutely while A2A antagonism makes a greater contribution following chronic caffeine administration due to the development of tolerance at A1 receptors. The symptomatic relief by adenosine receptor antagonists in Parkinson’s disease patients can be easily understood from the functional adenosine-dopamine interaction [**27**].

The selective adenosine A2A receptor antagonists have the ability to modulate the dopaminergic pathways implicated in disorders such as Parkinson’s disease and antipsychotic-induced extrapyramidal syndrome. Additionally, in vivo data observed in the primate support the large body of research in the rodent. Based on the primate studies, A2A receptor antagonists clearly have potential to treat numerous movement disorders in humans [**28**]. 

SCH58621, the A2A specific antagonist, demonstrated a higher degree of potency, maintaining its effectiveness at lower doses. Although CPT (8-cyclopentyltheophylline), the A1 specific antagonist, was able to show some reversal of catalepsy, the peripheral benefits of antagonism at the A2A site make this the most desirable adjunctive therapeutic approach. In addition, the A2A antagonist did not restore locomotion while the A1 antagonist did. While it was initially predicted that treatment with the A2A receptor antagonists would create the greatest restoration of movement in the animals, the results of the present different experiments indicate that the A2A antagonist was actually the least effective in restoring movement.

Adenosine and caffeine inhibited the development of haloperidol-induced orofacial dyskinesia supporting the involvement of adenosinergic receptor system in this debilitating movement disorder. Adenosine and caffeine possibly interact with different neurotransmitters such as dopamine. The involvement of adenosinergic receptor system in development of tardive dyskinesia was observed in many studies. Caffeine (A2A receptor antagonist) and adenosine also showed protective effects as it prevented the increase in oxidative damage markers in different brain regions mostly in striatum [**29**]. Adenosine receptor agonists and A2A receptor antagonists showed the protective effect against both excitotoxic effects of glutamate receptor activation as well as against damage induced by dopaminergic neurotoxin MPTP [**30**].

Adenosine and caffeine prevented the dopamine release followed by delayed development of supersensitivity to dopamine. Adenosine and caffeine dose dependently prevented the decrease of dopamine turnover in extracellular spaces [**31**].

Caffeine has greater potency and efficacy than theophylline to reverse the motor impairment caused by chronic but not by acute interruption of striatal dopaminergic transmission. This might be related to the neuroadaptive changes in the expression and coupling state of D2, A1 and A2A receptors occurring after DA depletion, which, in turn, could modify the relative affinity of methylxanthines for pre- and postsynaptic heteromers [**32**]. Given that theophylline showed less potency and efficacy than caffeine to reverse the motor impairment caused by chronic, but not acute, interruption of striatal dopaminergic transmission in rats, it is suggested that caffeine would provide more benefits than theophylline to improve the motor function in patients with Parkinson’s disease.

Caffeine, or other more selective adenosine A2A receptor antagonists, causes significantly less motor stimulation in dopamine D2 receptor knockout mice than in wild-type mice, which would indicate that the motor effects of caffeine, at least to some extent, are dependent on dopamine D2 receptors [**33**]. A2A receptor antagonists might also improve motor deficiency in dopamine D2 receptor knockout mice, and this indicates that caffeine can restore motor abnormalities in the absence of dopamine D2 receptors [**34**]. Thus, it would seem that one important role of D2 receptors in basal ganglia is to antagonize tonic A2A receptor stimulation. In line with this, it has been shown that adenosine A2A receptor knockout mice are partially protected against neuroleptic-induced catalepsy. The haloperidol induced swim disability was refurbished in l-dopa or caffeine pre-treated Swiss albino male mice due to restoration of dopamine concentrations [**35**].

A set of genetic KO models demonstrate that the A2A receptor exerts its neuronal activity in the striatum in a manner partially independent of D2 receptors. The D2 receptor-independent component of A2A receptors function is demonstrable at the behavioral (motor activity) as well as cellular (enkephalin mRNA expression) levels. Furthermore, A2A and D2 receptors produce opposite effects on encephalin mRNA expression, with A2AR-mediated stimulation of encephalin mRNA manifesting best when D2 receptor-mediated inhibition is removed. These results argue strongly for D2 receptor-dependent as well as D2 receptor-independent mechanisms of A2AR neural functions in vivo. Furthermore, they suggest that endogenous adenosine acting at striatal A2ARs may be most accurately viewed as a facilitative modulator of striatal neuronal activity rather than simply as an inhibitory modulator of D2 receptor neurotransmission [**36**].

It is conceivable that caffeine-induced dopamine D1 receptor down-regulation in the striatum and nucleus accumbens is the result of the removal of adenosine’s inhibitory effects on dopamine D1 receptors thereby producing overstimulation of these receptors. These data are consistent with some behavioral reports that tolerance to caffeine confers cross-tolerance to dopamine D1 or D2 receptor agonists alone, but not dopamine to D1 and D2 receptor agonists administered together or to D-amphetamine and cocaine [**37**, **38**]. However, it remains unclear why dopamine D2 receptor agonists would show cross-tolerance to caffeine if dopamine D2 receptors are not altered in response to chronic caffeine administration. There is evidence that dopamine receptor activation is probably the chief mechanism underlying the acute stimulatory effects of caffeine on locomotor activity. Even though haloperidol infusion concurrent with caffeine administration did not block the development of tolerance to caffeine-induced behavioral stimulation, there were clear changes in dopamine D1 receptors in the striatum, nucleus accumbens and prefrontal cortex in rats treated chronically with caffeine. Thus, specific changes in dopamine D1 receptor numbers are possible candidates for the mechanism underlying tolerance to the locomotor stimulant effects of caffeine [**39**].

**3.3. Caffeine and depression**

A2A adenosine receptor (AR) KO (knockouts) mice and wild-type mice injected with A2A AR antagonists were found to be less sensitive to ‘depressant’ challenges than controls [**40**], suggesting that blockade of adenosine A2A ARs might be an interesting target for the development of antidepressant agents. This antidepressant-like effect of selective A2A AR antagonists is probably linked to an interaction with dopaminergic transmission, possibly in the frontal cortex, since administration of the dopamine D2 receptor antagonist, haloperidol, prevents antidepressant like effects elicited by selective A2A AR antagonists in the forced swim test (putatively involving cortex), whereas it had no effect on stimulant motor effects of selective A2A AR antagonists (e.g. caffeine, putatively linked to ventral striatum) [**25**].

**3.4. Caffeine and schizophrenia**

No study so far has directly evaluated the influence of caffeine in schizophrenia, but there is growing evidence that adenosine dysfunction may contribute to the neurobiological and clinical features of schizophrenia [**41**]. Indeed, adenosine, via activation of A1 and A2A ARs, is uniquely positioned to influence glutamatergic and dopaminergic neurotransmission, two neurotransmitter systems that are mostly affected by the disease. It is possible that an adenosine inhibitory deficit may emerge, resulting in reduced control of dopamine activity and increased vulnerability to excitotoxic glutamate action in the mature brain. Interactions between A2A ARs and D2 receptors allow further opportunity for mutual modulation between the adenosine and dopamine systems [**42**]. These mechanisms could provide a rationale for an antipsychotic like profile of AR agonists, in particular A2A AR agonists, to promote a reduction in D2 receptor signaling [**42**], and A1 AR agonists to promote a reduction in dopamine release [**41**]. Indeed, dipyridamole, a well-known inhibitor of adenosine transporters, and therefore an enhancer of extracellular adenosine levels, may be of some therapeutic interest in schizophrenic patients [**43**].

Caffeine did not increase effortful choices, given the common use of caffeine to perform effortful tasks, and findings in the exercise psychology literature that caffeine reduces perceptions of effort [**44**]. However, this finding is in line with the literature data, and underscores the complex nature of A2A–DA interactions relating to motivation [**45**]. In rodents, adenosine A2A antagonists (unlike prototypic DA stimulants) do not increase preference for high effort options even though they reverse the effects of dopamine antagonism/depletion. In fact, in one article animals exhibited a trend towards fewer HC/HR (called “high cost/high reward” options) choices after caffeine (40 mg/kg), a dose that ameliorated DA antagonism [**45**]. The current study suggests that in humans as well, A2A antagonists do not increase effort in the same way as dopaminergic stimulants.The reasons for the differences between caffeine and amphetamine on this measure are not known. However, interactions between adenosine and dopamine are complex, and caffeine may act on multiple receptor types [**46**]. The striatum is thought to be a primary site of adenosine's action on effort-related processes [**47**], with both A1 adenosine receptors and A2A receptors present, co-localized with D1 and D2 receptors respectively [**46**]. Both D1 and D2 depletion/blockade affect exertion of effort, and A2A antagonists are able to reverse the effect of D2 antagonism on effort, while A1 antagonists are ineffective at ameliorating the effects of D1 or D2 antagonists [**45**, **48**]. Additional preclinical work suggests that the arousal-related effects of caffeine are exclusively mediated by A2A receptor function, as A2A-knockout mice exhibit no benefits from caffeine on wakefulness, while A1 knockouts show normal effects of caffeine. Interestingly, A2A knockouts are also resistant to the normal effects of D2 receptor antagonism on effort-based decision-making [**49**]. Although caffeine is a non-selective adenosine antagonist that influences both A1 and A2A receptors, it may be that its A2A effects are not as evident in this human paradigm. One future direction for this research is to examine the effects of caffeine in humans in the presence of a dopamine D2 antagonist, such as haloperidol. This would test the possibility that adenosine antagonist effects on effort are only visible during D2 depletion/antagonism.

**3.5. Caffeine and reward system**

The mechanism of action of caffeine indicates that adenosine receptors do not mediate caffeine’s appetitive and aversive effects. Caffeine has an atypical reward mechanism, independent of the dopaminergic system and the tegmental pedunculopontine nucleus, and provide additional evidence in support of a role for the dopaminergic system in aversive learning [**50**].

Moderate dose of caffeine does not strongly influence motivational behavior in healthy normal adults, although further research will be required to establish whether this result applies at different

caffeine doses, to different types of effort (cognitive vs. physical), during dopamine depletion, or in sub-populations with low baseline dopaminergic functioning [**51**].

Caffeine produced cocaine seeking is not mediated by adenosinergic A2 antagonism, but is contingent upon dopaminergic mechanisms. The nature of the interaction between caffeine and dopamine is not well understood, but warrants further investigation to determine whether they become more apparent following experience with cocaine self-administration [**52**].

Caffeine expectation induces dopaminergic placebo effects, and these effects are similar to previous findings with oral administration of caffeine. The results therefore suggest that caffeine and placebo caffeine may share some dopaminergic mechanisms of action [**53**].

**3.6. Caffeine - prolactin interaction**

In non-pregnant healthy women caffeine decreases plasma levels of prolactin. This action of caffeine may be mediated by an agonistic effect on dopamine neurotransmission in the central nervous system. Methylxanthines release catecholamines in the central nervous system, and several groups have reported that methylxanthines may also have a direct effect on dopaminergic effector systems. Since caffeine is the most widely used psychotropic agent in the world this effect and the possible decrease in milk production in lactating women needs further study, in view of potential reproductive and nutritional consequences [**54**].

**4. Conclusions and implications**

The current available scientific data support both dopaminergic and adenosinergic system pharmacodynamics interactions but also caffeine and dopaminergic system interactions. Caffeine can significantly improve Parkinson’s disease symptoms but also the extrapyramidal syndrome induced by antipsychotics via dopaminergic pathways. No study so far has directly evaluated the influence of caffeine in schizophrenia symptoms, but there is growing evidence that adenosine dysfunction may contribute to the neurobiological and clinical features of schizophrenia. Caffeine has also effects on the reward system but it seems that this effect does not involve dopaminergic system. Caffeine has some endocrine effects via dopaminergic system as decreasing the milk production in lactating women or other potential reproductive and nutritional consequences. Adenosinergic A2A receptors antagonists (Istradefylline, Preladenant, Vipadenant) show some promising therapeutic value in Parkinson’s disease. Due to wide use of caffeine, the research of interaction of this substance with different neurotransmitters system (adenosine, dopamine, GABA, glutamate) remains justified, especially in patients with schizophrenia, depression or Parkinson’s disease. 
